# Exploring the Implementation of Gamification as a Treatment Modality for Adults with Depression in Malaysia

**DOI:** 10.3390/medicina61081404

**Published:** 2025-08-01

**Authors:** Muhammad Akmal bin Zakaria, Koh Ong Hui, Hema Subramaniam, Maziah Binti Mat Rosly, Jesjeet Singh Gill, Lim Yee En, Yong Zhi Sheng, Julian Wong Joon Ip, Hemavathi Shanmugam, Chow Soon Ken, Benedict Francis

**Affiliations:** 1 Department of Psychological Medicine, Faculty of Medicine, Universiti Malaya, Kuala Lumpur 50603, Malaysia; akmalz@ummc.edu.my (M.A.b.Z.); ohkoh@um.edu.my (K.O.H.); jesjeet@um.edu.my (J.S.G.); u2004256@siswa.um.edu.my (L.Y.E.); u2004226@siswa.um.edu.my (Y.Z.S.); julian.wong@um.edu.my (J.W.J.I.); hemavathi19@um.edu.my (H.S.); soonken@um.edu.my (C.S.K.); 2 Department of Software Engineering, Faculty of Computer Science and Information Technology, Universiti Malaya, Kuala Lumpur 50603, Malaysia; hema@um.edu.my; 3 Department of Physiology, Faculty of Medicine, Universiti Malaya, Kuala Lumpur 50603, Malaysia; maziahmr@um.edu.my

**Keywords:** gamification, depression, digital therapy, tool, Malaysia

## Abstract

*Background and Objectives*: Depression is a leading cause of disability globally, with treatment challenges including limited access, stigma, and poor adherence. Gamification, which applies game elements such as points, levels, and storytelling into non-game contexts, offers a promising strategy to enhance engagement and augment traditional treatments. Our research is the first study designed to explore the implementation of gamification within the Malaysian context. The objective was to explore the feasibility of implementation of gamification as an adjunctive treatment for adults with depression. *Materials and Methods*: Focus group discussions were held with five mental health professionals and ten patients diagnosed with moderate depression. The qualitative component assessed perceptions of gamified interventions, while quantitative measures evaluated participants’ depressive and anxiety symptomatology. *Results*: Three key themes were identified: (1) understanding of gamification as a treatment option, (2) factors influencing its acceptance, and (3) characteristics of a practical and feasible intervention. Clinicians saw potential in gamification to boost motivation, support psychoeducation, and encourage self-paced learning, but they expressed concerns about possible addiction, stigma, and the complexity of gameplay for some patients. Patients spoke of gaming as a source of comfort, escapism, and social connection. Acceptance was shaped by engaging storylines, intuitive design, balanced difficulty, therapist guidance, and clear safety measures. Both groups agreed that gamification should be used in conjunction with standard treatments, be culturally sensitive, and be presented as a meaningful therapeutic approach rather than merely as entertainment. *Conclusions*: Gamification emerges as an acceptable and feasible supplementary approach for managing depression in Malaysia. Its success depends on culturally sensitive design, robust clinical oversight, and seamless integration with existing care pathways. Future studies should investigate long-term outcomes and establish guidelines for the safe and effective implementation of this approach. We recommend targeted investment into culturally adapted gamified tools, including training, policy development, and collaboration with key stakeholders to realistically implement gamification as a mental health intervention in Malaysia.

## 1. Introduction

Depression remains one of the most pressing public health challenges worldwide. It affects over 300 million people globally and is a leading contributor to disability, loss of productivity [1], and is 30 times more likely to lead to suicide [2,3]. In Malaysia, recent figures from the National Health and Morbidity Survey (2023) indicate an upward trend in the prevalence of major depressive disorder, now estimated at 4.6% nationally, with some urban regions such as Putrajaya reporting alarmingly high rates of up to 29% [4]. The sharp increase in reported cases over the past five years reflects not only a growing mental health burden but also the urgent need for new strategies in detection, treatment, and sustained engagement.

While pharmacological and psychological treatments are well established for managing depression, their real-world application is often laden with challenges, as reported in many studies [5,6,7]. These challenges include limited availability of mental health professionals, stigma surrounding help-seeking, and practical barriers such as time constraints, cost, and transportation. Even among those who begin treatment, poor adherence and early dropout are prevalent [8]. Many patients struggle with low motivation, emotional withdrawal, and cognitive fatigue, all of which diminish their ability to engage meaningfully with therapy. The discourse surrounding unmet needs in the treatment of depression must expand beyond simply addressing treatment gaps and also include issues about treatment delivery gaps [9]. Thus, gamification plays a crucial role in bridging this delivery gap in the treatment of depression.

Gamification, which employs game elements such as points, levels, feedback, and storytelling in non-game contexts, has garnered increasing attention as a strategy to enhance motivation and engagement in healthcare [10]. The term gamification in medicine refers to the integration of game-like elements (points, rewards, levels) into clinical settings to be used as an intervention. This terminology differs slightly from that of serious games, which are games developed exclusively for educational or therapeutic purposes. In summary, the key distinction lies in scope and integration, whereby gamification modifies existing structures to make them more interactive, while serious games are explicitly designed with intervention in mind [11,12,13,14]. In mental health, various gamified interventions have been developed internationally to support the treatment of depression [15]. These include serious games like SPARX in New Zealand [16], which provides cognitive behavioural therapy (CBT) through an interactive fantasy setting; casual video games that involve problem-solving, rhythm, or adventure tasks to improve mood and reduce stress; and CBT-based mobile applications such as SuperBetter [17] and Depression Quest [18], which incorporate behavioural activation through structured missions and journaling tasks [10]. Some platforms also utilise mood-tracking tools with game-like features, such as reward badges or visual feedback, to reinforce self-awareness and track progress over time [19].

Common across these interventions is the strategic use of incentives to maintain engagement. These may include levelling up, earning virtual rewards or trophies, unlocking new game environments, narrative progression linked to symptom improvement, or even simple affirmation messages that provide emotional reinforcement [20]. SPARX, for example, is a role-playing game that delivers core principles of cognitive behavioural therapy through an engaging fantasy world. Players complete quests that mirror real-life emotional challenges, learning coping strategies such as problem-solving, relaxation techniques, and cognitive restructuring processes. Similarly, SuperBetter integrates gamified elements into a structured narrative to promote resilience and self-efficacy. Users design their quests and enlist allies, thus tracking progress towards personal goals. Studies on SuperBetter and SPARX have reported improvement in engagement, cognition and depressive symptoms [17,21]. These serious games work not merely through entertainment but by embedding evidence-based psychological interventions within compelling, interactive experiences. These mechanisms are particularly effective in addressing core depressive features like anhedonia, low motivation, and social withdrawal [15,22,23,24]. By translating therapeutic goals into small, achievable challenges, gamified tools render the treatment experience more manageable and motivating, especially for users who may find conventional therapy overwhelming or challenging.

In Malaysia, the application of gamification in mental healthcare is still in its infancy. Despite high rates of mobile phone usage, digital therapeutics remain largely absent from routine psychiatric practice [25]. Public attitudes towards gaming are often ambivalent, with concerns about its perceived frivolity, potential for addiction, and lack of legitimacy as a health tool [26]. At the same time, a clear unmet need remains for interventions that are more accessible, less stigmatising, and capable of reaching underserved populations [27]. This scenario is particularly true in rural or resource-limited areas where psychiatric services are scarce and for patients who may be reluctant or unable to engage in traditional talk therapy. Gamification may offer a novel entry point, one that aligns with users’ habits and preferences while embedding therapeutic content in an engaging and approachable format [28].

This study aims to address that gap by exploring the feasibility and practicality of using gamification as a treatment tool for adults with depression in Malaysia. We utilised qualitative methods to engage healthcare professionals and patients in structured discussions, thereby better understanding their perspectives, preferences, and concerns. By capturing these voices, we aim to investigate the development of culturally relevant and clinically appropriate gamified mental health interventions that could be employed in routine care.

## 2. Materials and Methods

### 2.1. Study Design

This study employed a qualitative focus group discussion methodology to examine two levels of perspective. The first perspective assessed the comprehension and knowledge of gamification among service providers, specifically the professional group. The second level evaluated the consumer or client’s experience in gaming and their expectations of the game as a treatment for depression.

The clinicians’ group was selected based on purposeful sampling following these criteria:

- Registered medical practitioners in mental health.
- Experience with gaming and the concept of gamification. To identify this group of patients, a preliminary interview was conducted to assess their suitability.

The selected medical practitioners were MP1, MP2, MP3, MP4, MP5.

For the patient group, ten patients were recruited by purposeful sampling with the following criteria:

### 2.2. Inclusion Criteria

- A registered patient in the Psychological Medicine clinic, University Malaya Medical Centre (UMMC).
- Aged 18–55 years old.
- Able to read and understand English.
- Had a clinical diagnosis of Major Depressive Disorder based on the Diagnostic and Statistical Manual for Mental Disorders (DSM-5).

- Patient Health Questionnaire-9 (PHQ-9) score: 4–15.
- Patients have been on pharmacological standard-of-care treatment for their depression during the duration of intervention.
- The pharmacological standard of care treatment has been present for more than 8 weeks and unchanged for at least 4 weeks at the point of recruitment.

### 2.3. Exclusion Criteria

- Another mental illness diagnosis besides Major Depressive Disorder.
- Presence of suicidality.
- Refusal to join the study.

The patient samples were then labelled as P1, P2, P3, P4, P5, P6, P7, P8, P9, P10.

The patients were grouped into two and interviewed on different occasions until the point of saturation was reached.

Each interview lasted at least 45 min, and participants were asked to share their perceptions of the points highlighted during the interview. The following were the questions asked of the participants:

i Clinician group
  - Awareness of the gamification as a treatment option for depression: benefits, side effects, concerns.
  - Acceptance and adherence to gamification as a treatment for depression.
  - The practicality of gamification as a treatment: funds or resources available, applicable platforms, devices that will be used, game development, tools in the gamification that help treatment, number of users/players.
  - Feasibility of gamification as a treatment: which client group, platform, prescribed frequency, elements in therapy needed, timing to engage in the treatment, and side effects.
  - Inclusion of gamification as the standard of care in local practice.
ii Patient group
  - Elements in the game that they like: themes, reason for playing, time spent, device used.
  - Practicality of the treatment: game preference, which will make users adhere to the treatment, preferred device and platforms, and side effects of gamification as a treatment.
  - Feasibility of gamification as a treatment: factors that make gamification accepted by users, timing, and place to engage in gamification as a treatment.

All patients had a clinically confirmed diagnosis of Major Depressive Disorder, and the severity of the condition was assessed using the PHQ-9 and the Generalised Anxiety Disorder-7 (GAD-7) scales. All the recruited patients were also from an urban setting, owned smartphones, and were familiar with gaming.

### 2.4. PHQ-9

The Patient Health Questionnaire-9 (PHQ-9) is a widely used self-administered screening tool for assessing the severity of depression. Comprising nine items that align with the diagnostic criteria for major depressive disorder in the DSM, it allows for quick evaluations in both clinical and research settings. Each item is scored from 0 (not at all) to 3 (nearly every day), with total scores ranging from 0 to 27, indicating minimal to severe depression. The PHQ-9 has been validated across diverse populations and is valued for its brevity and strong psychometric properties [29]. It has also been validated in the Malaysian population, demonstrating good reliability and validity in local settings [30].

### 2.5. GAD-7

The Generalised Anxiety Disorder-7 (GAD-7) is a brief, self-administered questionnaire designed to screen for and assess the severity of generalised anxiety disorder. It consists of seven items, each rated on a scale of 0 (not at all) to 3 (nearly every day), with total scores ranging from 0 to 21. The GAD-7 is widely used in clinical practice and research due to its strong validity, reliability, and ease of use [31]. It has also been validated for the Malaysian population, demonstrating good psychometric performance in local primary care and clinical settings [32].

Data from the focus groups were analysed using thematic analysis, following Braun and Clarke’s six-phase framework. Two researchers independently coded the transcripts. Initial codes were generated inductively and then grouped into potential themes. Discrepancies were discussed and resolved through consensus. Themes were refined and named based on prevalence and relevance to the research question.

This study received ethical approval from the University Malaya Medical Centre Ethical Committee (MECID: 202319-11957). All participants (clinicians and patients alike) provided written informed consent prior to participation. Data were anonymised using coded identifiers to ensure confidentiality.

## 3. Results

### 3.1. Participants’ Demographic Data

This study analysed demographic and psychological parameters of 15 participants, coded as MP1–5 and P1–P10. The key findings are summarised in Table 1:

The study included 15 participants: 5 professionals (mean age = 35 years) and 10 patients with depression (mean age = 31 years). The majority were female (73.3%) and lived in urban areas (100%). Most participants resided with family (86.7%) and were single (66.7%). The ethnic distribution comprised Malays (46.7%), Chinese (33.3%), along with a smaller proportion of Indians and others. All participants owned smartphones and reported playing video games. Among patients, PHQ-9 scores indicated predominantly mild to moderate depression (mean score: 10.27), while GAD-7 scores revealed that all patients exhibited minimal to mild anxiety (mean score: 9.75). Patients generally had lower income levels, with 80% earning less than RM5000 per month, contrasting with the higher earnings of professionals. The sample reflected a tech-savvy, urban population with active gaming experience and clinically significant symptoms of depression and anxiety.

Three main themes were identified from the focus group discussions: (1) understanding of gamification as a treatment option, (2) conditions that influence acceptance, and (3) characteristics of a practical and feasible game-based intervention for depression. These themes emerged across both professional and client groups.

### 3.2. Understanding of Gamification as a Treatment Option

Participants displayed varying levels of familiarity with gamification. Professionals recognised the potential value of gamification in enhancing motivation, tracking treatment progress, and supporting psychoeducation. One practitioner noted, “We can focus on certain motivational elements that can help to engage them better, improve their negative thinking, and give them more motivation to engage” (MP1). Another observed that gamification might empower patients by allowing them to practise skills independently and at their own pace: “… psychotherapy elements that you want to incorporate into your patients so that they can practice it in their own space and their own time outside the clinical setting” (MP4).

Despite this, several clinicians expressed reservations. Some were concerned about the risks of gaming addiction, the stigma associated with gaming in Malaysian society, and the complexity of gameplay for patients with reduced cognitive functioning. As one put it, “Games are not accepted here” (MP1), while another said, “There are too many steps to learn. Not for the depressed individual” (MP4).

Patients, on the other hand, spoke positively about gaming. For many, gaming provided escapism, social connection, and emotional regulation. One patient explained, “It allows me to enter a different world… it makes me become immersed in a world other than the one we live in” (P4). Others mentioned that games helped them bond with friends or cope during difficult times: “I play with my friends… It is not a multiplayer game, and I just invite them to play along with me” (P1). Several patients acknowledged using games as a personal coping strategy.

### 3.3. Conditions That Influenced Acceptance

Factors influencing the acceptance of gamified treatment fell into three categories: individual readiness, game-related features, and therapist involvement. Patients emphasised that preferences in game design, such as engaging storylines, graphics, and predictability, contributed to sustained interest. “The storytelling… it is a constant story, so I keep returning to it to learn more” (P4). Themes of strategy, role-play, and exploration were particularly appealing.

The significance of an intuitive interface and achievable difficulty was also emphasised. “If it is too hard, it is not appealing… but if it is too easy, also the same” (P1). Patients valued customisable avatars and reward mechanisms, particularly those that could unlock new content or reflect their achievements.

The professionals’ concerns centred around selecting suitable patients, providing therapist training, and integrating safety features. One clinician stated, “Not everybody will accept it… there is a risk people may see it in a very negative light” (MP1). Another highlighted the importance of “building patient motivation and confidence first, then their engagement with the therapy” (MP2).

### 3.4. Characteristics of a Practical and Feasible Intervention

Both groups shared perspectives on what would render gamification practical and culturally relevant. Clinicians emphasised that gamification should be introduced once patients achieve stability, ideally complementing pharmacotherapy: “Serious gaming therapy should be introduced following the initial phase of pharmacological treatment” (MP2). For convenience and privacy, patients favoured playing on smartphones, stating, “I prefer mobile… Not everybody wants others to know that they are playing a game for mental health” (P5).

Safety and moderation were common concerns. Due to risks such as cyberbullying, single-player formats were preferred over online multiplayer modes: “I cannot tolerate the toxicity of online gaming anymore” (P1). Suggested safety features included time restrictions, session locks, and monitoring tools. The content and design of the game were also discussed. Participants recommended incorporating themes from daily life, using bright visuals, adding background music, and developing emotional storylines. Therapists wanted games to track patient progress, reinforce positive behaviour, and offer feedback loops: “You can assess the patient’s response to treatment based on game progress” (MP4).

Finally, participants noted that gamification should incorporate elements of cognitive behavioural therapy, psychoeducation, or mindfulness and be flexible enough to be utilised during leisure time: “You can do it during your free time… two to three hours should be fine” (MP2).

Clinicians recognised the value of gamification in addressing everyday challenges in depression, such as low motivation and poor adherence, and appreciated the potential for structured reward goals. Setting and personalising avatars to help patients remain engaged raised concerns about selecting the right patients, supervising their use, and managing the risks related to gaming-related harm: “Gaming characters are interesting but I wonder if it applies to all patients. Avatars must be chosen carefully. We do not want to create addiction issues later” (MP5). Patients shared a different yet equally thoughtful perspective. Many have already utilised games for emotional escape and comfort, especially during difficult times. They felt that story-driven games with relatable characters and music could help improve their mood and foster a sense of control: “I do play video games when I feel down, sometimes I choose fast-paced games to help lift my mood. The music of the game is important too as it helps sets the tone of the game” (P7). Interestingly, both groups agreed that for gamification to be effective, it needed to be reframed, not just as “just a game”, but as a meaningful and therapeutic experience.

Overall, participants from both groups were cautiously optimistic about using gamification for depression. They recognised its motivational potential, ease of access, and adaptability, but emphasised the importance of appropriate design, therapist involvement, and safety monitoring.

## 4. Discussion

Our study provides novel insights into the perception of gamification and its utility as a mental health tool within the Malaysian context. Gamification possesses real potential to make mental healthcare more engaging, accessible, and responsive to patients’ needs [28]. However, this will only occur if the tools are culturally appropriate, clinically grounded, and delivered with care [32,33]. This study contributes to the evidence that, with the proper foundation, Malaysia is prepared to explore what gamified therapy can offer. Nonetheless, one should proceed cautiously, as gamification is not a substitute for professional therapy and may not be suitable for everyone [34]. Some participants expressed concern about becoming overly immersed in the games or losing interest if the novelty wore off. These concerns are valid. Any serious application of gamification in mental health must be accompanied by monitoring and support, just like any other treatment [35].

One of the most significant takeaways from this study is that gamification could help reach patients who are underserved by the current system. This approach might work best for those in recovery or those who find it difficult to attend regular sessions due to distance, cost, or stigma. The caveat, however, is that for this method to succeed, the design must be patient-centric, with well-designed safety features such as time limits, and it should come with clear guidance on its use. Thus, training therapists to incorporate these tools into their practice is essential, as is allowing space for feedback and adaptation [36]. Digital mental health tools such as SPARX and SuperBetter have shown that well-designed therapeutic games can improve outcomes for depression in a nuanced and welcoming fashion, thus removing the weight of stigma [37]. Most of these tools were created in Western countries with different cultural expectations and more established digital health systems. Our findings suggest that these models cannot simply be imported; settings, dialogue, and character roles must reflect local languages and everyday experiences to feel authentic and build trust.

To make sense of our findings, it is helpful to highlight a couple of psychological theories, namely the Technology Acceptance Model (TAM) [38,39] and the Self-Determination Theory (SDT) [40]. TAM suggests that people are more likely to accept and use technology if they find it valuable and easy to navigate. This model aligns with what our patients highlighted during the focus group discussions, namely, client-friendly features within the game, simple navigation, and mobile accessibility, which made the idea of a gamified mental health tool more appealing. Meanwhile, in the SDT framework, people are motivated when their needs for autonomy, competence and social connection are met. Participants echoed these needs through their preference for self-paced games (autonomy), clear feedback and goals (competence), and emotionally meaningful gameplay (relatedness). In short, fulfilling these psychological needs translates to better acceptability and real-world impact.

In Malaysia, there remains a strong preference for traditional care and time-honoured mental health interventions [41]. Our study found that gaming is often perceived as a distraction or vice rather than a means to support healing. Accordingly, it was understandable that patients favoured private, offline play, while professionals sought structure, oversight, and a gradual rollout of gamified features. Cultural factors, including the importance of family, modesty, and the avoidance of shame, likely influenced the prioritisation of discretion, personalised content, and therapist support.

Whilst gamification is widely recognised for boosting motivation and engagement, it also presents risks. Research from other countries has indicated that specific game mechanics, such as leaderboards and public rankings, can occasionally foster negative behaviours like cyberbullying and social exclusion [42]. A growing concern exists that gamified systems, particularly those that replicate reward structures found in video games, may contribute to addictive behaviours [26]. Malaysia’s collectivist culture, which values group harmony and discourages open conflict, may help mitigate some of the risks associated with aggressive online behaviour. However, the rapid increase in digital media usage and the relatively limited focus on digital wellbeing policies might render young people particularly susceptible to issues such as online addiction. This paradigm underscores the importance of thoughtful design that strikes a balance between user engagement and safeguards to protect users.

While global studies, such as SPARX and SuperBetter, demonstrate how serious games can support mental health, our findings are nuanced by incorporating the voices of Malaysian participants. Unlike the Western demographic who may be more open to digital mental health tools, many of our participants expressed hesitation due to stigma, privacy concerns, and the social acceptability of those tools. For our patients, key considerations for the successful implementation of gamification included having games in their local language, incorporating culturally relevant themes, and receiving guidance from therapists. Similar trends have been observed in neighbouring countries. In an Indonesian study, researchers found that gamified learning applications for students need to be adapted with culturally relevant visuals and stories to be effective [43]. In Thailand, the successful participation in a school-based pilot study involved strong parental guidance and the incorporation of the local Thai language into the gamified software [44]. A Vietnamese study studied the use of gamified Cognitive Behavioural Therapy (CBT) techniques for university students with mild depression. It found that assimilation of the local language, cultural symbolism and family-inclusive narratives were key to user engagement and trust [45]. These examples reflect a shared regional theme: for gamification to succeed in the region, it must be culturally and linguistically appropriate while retaining family-centric values.

Our study offers new insights into how gamification could support depression treatment in Malaysia. Through focus group discussions with clinicians and patients, we discovered that while the idea was generally well-received, there were significant concerns regarding its practical application. Three themes emerged: the concept was promising yet unfamiliar, its success would hinge on careful design and delivery, and most participants believed it should complement, rather than replace, existing treatment options. Our study has its limitations. The principal limitation is its small sample size and the homogeneity of the population, which was urban, tech-savvy, and already familiar with gaming. As such, our findings may not be generalisable to rural populations or those less familiar with gaming. Secondly, the qualitative nature of the study limits the ability to make causal inferences. Further research involving larger, more diverse samples and possibly mixed methods is needed to validate and expand upon these findings.

## 5. Conclusions

This study is the first of its kind to explore the role of gamification in the treatment of depression. Its successful implementation depends on cultural tailoring, clinician involvement, and robust design frameworks that integrate psychoeducational and behavioural activation principles. With thoughtful adaptation, gamification has the potential to bridge access gaps and enhance treatment engagement in Malaysian mental healthcare. Future research should examine the long-term effects of gamification on mental health and social behaviours, paying attention to cultural factors. It should also include more diverse populations to capture a broader range of perspectives. To support the integration of gamification into mental health treatment, the authors recommend the development of culturally tailored gamified interventions that incorporate local narratives and language options (Malay, Tamil, Mandarin). Thus, collaboration with game developers, mental health professionals and policymakers is crucial to ensure clinical safety, accessibility and scalability. One interesting suggestion is to design pilot studies at both urban and rural clinics to test the feasibility of gamification in real-world settings. Furthermore, as clear policy is lacking in the field of digital health in Malaysia, clear guidelines must be established to ensure successful and safe implementation of gamification in a clinical setting. Additionally, creating versions of a gamified intervention in various vernacular languages in Malaysia will improve usability and accessibility to this novel intervention.

## Figures and Tables

**Table 1:** Socio-demographic characteristics of adults with depression.

Socio-Demographic	Professional (*n* = 5)		Patient *(n* = 10)		Total	
	*n* (%)	Mean (SD)	*n* (%)	Mean (SD)	*n* (%)	Mean (SD)
**Age**		35 (2.550)		31 (7.379)		32.33 (6.377)
**Gender**						
Male	2 (40)		2 (20)		4 (26.7)	
Female	3 (60)		8 (80)		11 (73.3)	
**Residential Area**						
Urban	5 (100)		10 (100)		15 (100)	
**Living with**						
Family	4 (80)		9 (90)		13 (86.7)	
Alone	1 (20)		1 (1)		2 (13.3)	
**Marital Status**						
Single	3 (60)		7 (70)		10 (66.7)	
Married	2 (40)		3 (30)		5 (33.3)	
**Ethnicity**						
Malay	2 (40)		5 (50)		7 (46.7)	
Chinese	2 (40)		3 (30)		5 (33.3)	
Indian	1 (20)		0		1 (6.7)	
Other	0		2 (10)		2 (13.3)	
**Religion**						
Islam	2 (40)		7 (70)		9 (60)	
Buddhism	1 (20)		2 (20)		3 (20)	
Christian	1 (20)		1 (10)		2 (13.3)	
Not Religious	1 (20)		0		1 (6.7)	
**Income Group**						
<RM 2500	0		4 (40)		4 (26.7)	
RM 2500–RM 5000	0		4 (40)		4 (26.7)	
RM 5000–RM 8000	3 (60)		1 (10)		4 (26.7)	
>RM 8000	2 (40)		1 (10)		3 (20)	
**Possession of a phone**						
Yes	5 (100)		10 (100)		15 (100)	
**Playing video games**						
Yes	5 (100)		10 (100)		15 (100)	
**PHQ 9 score**						10.27 (7.314)
**Category of PHQ 9**						
No Depression	3 (60)					
Mild Depression	2 (40)					
Moderate Depression	0					
Moderately Severe Depression	0					
Severe Depression	0					
**GAD-7 score**						9.73 (5.535)
**Category of GAD-7**						
Minimal Anxiety	4 (80)					
Mild Anxiety	1 (20)					
Moderate Anxiety	0					
Severe Anxiety	0					

## Data Availability

The original data presented in this study are openly available at Figshare at the following doi:10.6084/m9.figshare.29225372 or at https://figshare.com/s/01d288b504e8f9a9512b(accessed on 31 July 2025).
